# Effect of joint roughness coefficient and size on shear and characteristic strengths of structural planes

**DOI:** 10.1371/journal.pone.0265916

**Published:** 2022-04-21

**Authors:** Gaojian Hu, Juan Zhang, Wenxu Liang, Jie Wang, Jianli Hu, Long Wang

**Affiliations:** 1 School of Civil Engineering, Shaoxing University, Shaoxing, Zhejiang, China; 2 Key Laboratory of Rock Mechanics and Geohazards of Zhejiang Province, Shaoxing, Zhejiang, China; 3 Zhejiang Rock Mechanics and Geological Hazard Laboratory, Shaoxing, Zhejiang, China; 4 Shenyang Research Institute, China Coal Technology and Engineering Group, Fushun, Liaoning, China; 5 State Key Lab of Coal Mine Safety Technology, Fushun, China; 6 Shangdong Gold Design Consulting Co., Ltd., Yantai, Shandong, China; China University of Mining and Technology, CHINA

## Abstract

Joint roughness coefficients (JRCs) influence the shear and characteristic strengths of structural planes; however, the relationship model of this influence is yet to be derived. This study investigates 11 numerical simulation programs using a realistic failure process analysis software. The influence of size and JRC on the shear strengths of the structural planes was studied. The stress-strain curves of different JRCs and their sizes were analyzed. Mathematical models of the shear strength of structural planes and JRC and sizes were formulated and proposed, and their expressions were obtained. Moreover, mathematical models of JRC and the characteristic size and strength of the structural planes were established.

## Introduction

Rock mass is a complex geological body with randomly distributed joints and other defects. These unique structures cause the rock mass to exhibit size effect [1]. Rock mass stability depends on structural planes considerably, and shear strength is an important mechanical property [2,3]. This strength also manifests as the size effect. The joint roughness coefficient (JRC) also influences the mechanical properties of the rock mass considerably.

The rock size effect has been of significant research interest worldwide. For instance, Frossard et al. [4] proposed a method for calculating the shear strength based on the size effect considering fracture mechanics. To study the influence of dimensionality on rock shear strength, Nini et al. [5] used a three-dimensional (3D) distinct element code software to implement direct shear test simulations on rock blocks of different sizes. Scholars have also investigated the effects of size on smooth jointed rocks. For example, Wang et al. [6] studied the influence of block size on shear strength parameters. Researchers have also examined the effect of size on rough jointed rocks. For example, Huilin et al. [7] conducted direct shear tests on specimens of different sizes with serrated structural planes. Chen et al. [8] used 3D printing technology to fabricate structural plane molds. In addition, they conducted numerical experiments to determine the size effect on the shear strength of structural planes using particle flow code (PFC) software. Liu et al. [9] established a model using the PFC software and studied the effect of joint length on peak shear strength according to the Barton curve. Han et al. [10] investigated the shear mechanical performance of cement grouts cured for different periods. However, these studies did not consider the effect of size on the JRC. The relationship between the JRC and size, and its effect on the shear strength, was not derived.

The structural planes of rock blocks come into contact at the joints; hence, the mechanical properties of a rock unit differ from those of the entire rock mass considerably. The JRC affects the real contact area, which plays a significant role in the shear mechanical properties of the structural planes. For example, Ban et al. [11] proposed a model of rock joint peak shear strength based on roughness parameters and shear test results. Zhang et al. [12] employed direct shear test data to study the influence of roughness on shear strength. Scholars have used shear flow tests to study the influence of the JRC on shear strength. For example, Wang et al. [13] conducted a shear flow test on two rough cracks to study the effect of fracture plane roughness on the shear of rough wall rock cracks. Scholars have studied the relationship between the JRC and shear strength based on field tests. For instance, Ding et al. [14] proposed a relationship between the peak shear strength and JRC through a direct shear test. Fereshtenejad et al. [15] studied the influence of JRC on the shearing mechanics of 3D-printed samples through direct shear experiments. Yang et al. [16] conducted triaxial compression tests on rock specimens with different JRCs to investigate the relationship between peak strength and JRC. Scholars have performed several studies on the influence of the JRC on shear strength. Some of these investigations were related to the size effect. Nevertheless, studies on the relationship between the rock mass size and the shear strength of structural planes are limited.

The physical and mechanical parameters of the rock vary with the rock size. At a certain critical size, the mechanical parameters of the rock cease to vary with the increase in rock size. This is called the characteristic size of the representative elementary volume (REV) of a rock, and the rock strength corresponding to this size is called the characteristic strength. For example, Fan et al. [17] used realistic failure process analysis (RFPA) software to study rock size effects and characteristic unit bodies under different loading methods. In their studies, scholars have also derived the REV. For example, Pengpeng et al. [18] considered fracture roughness and introduced a new damage coefficient for calculating the REV size. Zhao et al. [19] performed a discontinuity scan to obtain the REV size of the characteristic dimension. The test was conducted at the Shirengou Iron Mine. Wang et al. [20] estimated the characteristic dimensions of the rock mass REV size through radial and unidirectional flow configurations. Huang et al. [21] estimated the REV size of a fractured rock mass based on the geological strength index. Some scholars have adopted various methods to determine the REVs of fractured rock masses. For example, Mahnaz et al. [22] determined the REV and rock deformability using Monte Carlo method. Chen et al. [23] introduced two new methods for determining the REV of fractured rock masses based on the concepts of hydropower station engineering. Zhang et al. [24] determined the REV of a fractured rock mass based on seepage theory. The abovementioned studies have obtained the characteristic size of rocks using different methods. However, a mathematical model representing the characteristic size and JRC and the characteristic shear strength and JRC has not been established.

In this study, 11 numerical simulation programs were formulated using RFPA. The influence of (i) JRC and (ii) size on the shear strength of structural planes was investigated, and the stress–strain curves of different JRCs and sizes were analyzed. Mathematical models of the shear strength of the structural plane, JRC, and size were established. The mathematical models of JRC, characteristic size of structural planes, and characteristic shear strength of structural planes were developed.

### Numerical simulation program and simulation parameter setting

The software used in this simulation is RFPA, which employs a numerical calculation method for simulating inhomogeneous materials, developed based on the finite element theory and statistical damage theory. The method accounts for the non-uniformity and randomness of materials and incorporates the statistical distribution assumptions of material properties into the finite element method. The mechanical properties of discretized meso-primitives are assumed to obey a certain statistical distribution law. Consequently, the relationship between meso- and macro-medium mechanical properties was established. The method is described by the Weibull statistical distribution function given by

$$\varphi (\alpha )=\frac{m}{\alpha_{0}}\cdot {\left(\frac{\alpha }{\alpha_{0}}\right)}^{m-1}\cdot e^{-{\left(\frac{\alpha }{\alpha_{0}}\right)}^{m}},$$ (1)

where α is a basic mechanical parameter of the material medium (e.g., elastic modulus, strength, Poisson’s ratio, and weight), α_0_ is the average value of α, m is the property parameter of the distribution function, whose physical meaning reflects the uniformity of the material medium, and φ(α) is the statistical distribution density of the material’s elementary mechanical property.

#### Numerical simulation program and model formulation

The numerical simulation contained two research contents. The first pertains to the influence of JRC on the shear strength of structural planes, and the second relates to the size effect on the shear strength of structural planes. For the first research content, six sets of numerical simulation programs were developed. The model sizes were 200 × 200, 400 × 400, 600 × 600, 800 × 800, 1000 × 1000, and 1200 × 1200 mm^2^. Each set of simulation programs contained five types of JRC conditions; the JRCs were 1.7, 2.7, 3.7, 4.7, and 5.7. The numerical simulations of shear were conducted under each working condition. For the second research content, five sets of numerical simulation programs with JRC values of 1.7, 2.7, 3.7, 4.7, and 5.7 were developed. Each set of simulation programs contained six types of model size working conditions; the model sizes were 200 × 200, 400 × 400, 600 × 600, 800 × 800, 1000 × 1000, and 1200 × 1200 mm^2^. The numerical simulations of shear were performed under each working condition. The specific numerical simulation programs are presented in Table 1, Where l is the rock size, and r is the JRC.

**Table 1:** Summary of research plans and working conditions.

Simulation program	JRC	Summary of research plans and working conditions					
		Program 1	Program 2	Program 3	Program 4	Program 5	Program 6
		*l* = 200 mm	*l* = 400 mm	*l* = 600 mm	*l* = 800 mm	*l* = 1000 mm	*l* = 1200 mm
**Program 7**	*r* = 1.7	1.7×200	1.7×400	1.7×600	1.7×800	1.7×1000	1.7×1200
**Program 8**	*r* = 2.7	2.7×200	2.7×400	2.7×600	2.7×800	2.7×1000	2.7×1200
**Program 9**	*r* = 3.7	3.7×200	3.7×400	3.7×600	3.7×800	3.7×1000	3.7×1200
**Program 10**	*r* = 4.7	4.7×200	4.7×400	4.7×600	4.7×800	4.7×1000	4.7×1200
**Program 11**	*r* = 5.7	5.7×200	5.7×400	5.7×600	5.7×800	5.7×1000	5.7×1200

In the JRC acquisition process, the first step is to use a structural surface contour sampler [25] to draw the contour of an on-site structural surface. Second, the drawn structural surface profile is converted into a digital file using data scanning, extraction, and analysis techniques [26]. In this study, the JRC values of the structural surfaces were calculated using algorithmic programming. Finally, the digital files were imported into the simulation software. The structural surface profile used in this study was taken from the slope of Zhangao Mine in Zhangao Village, Shengzhou City, Zhejiang Province, China.

**Fig 1:**
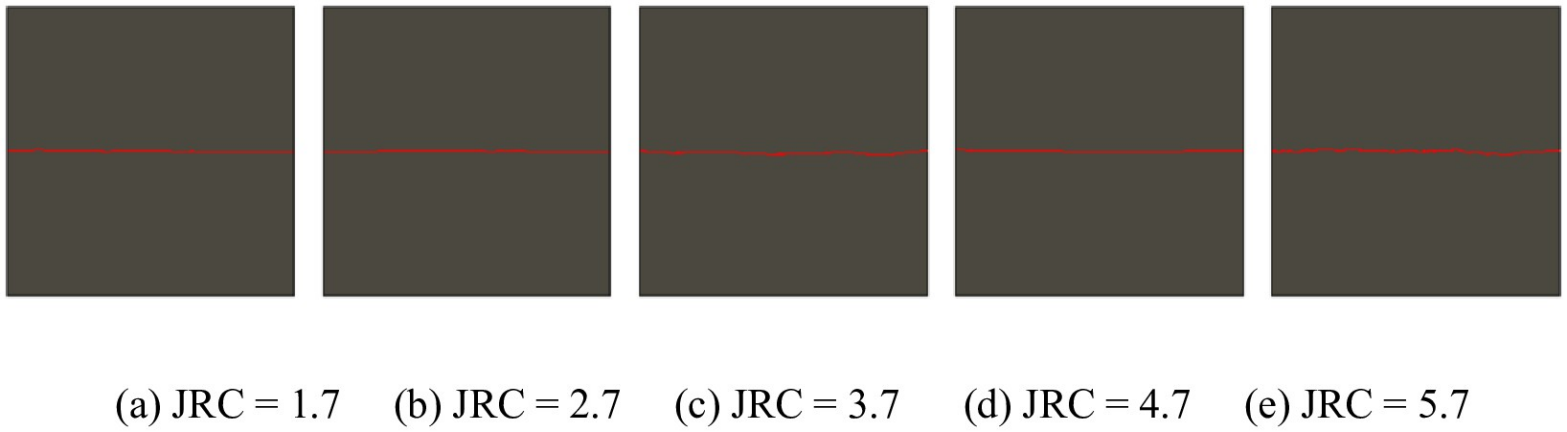
Rock model with different JRCs when size is 200 × 200 mm^2^.

**Fig 2:**
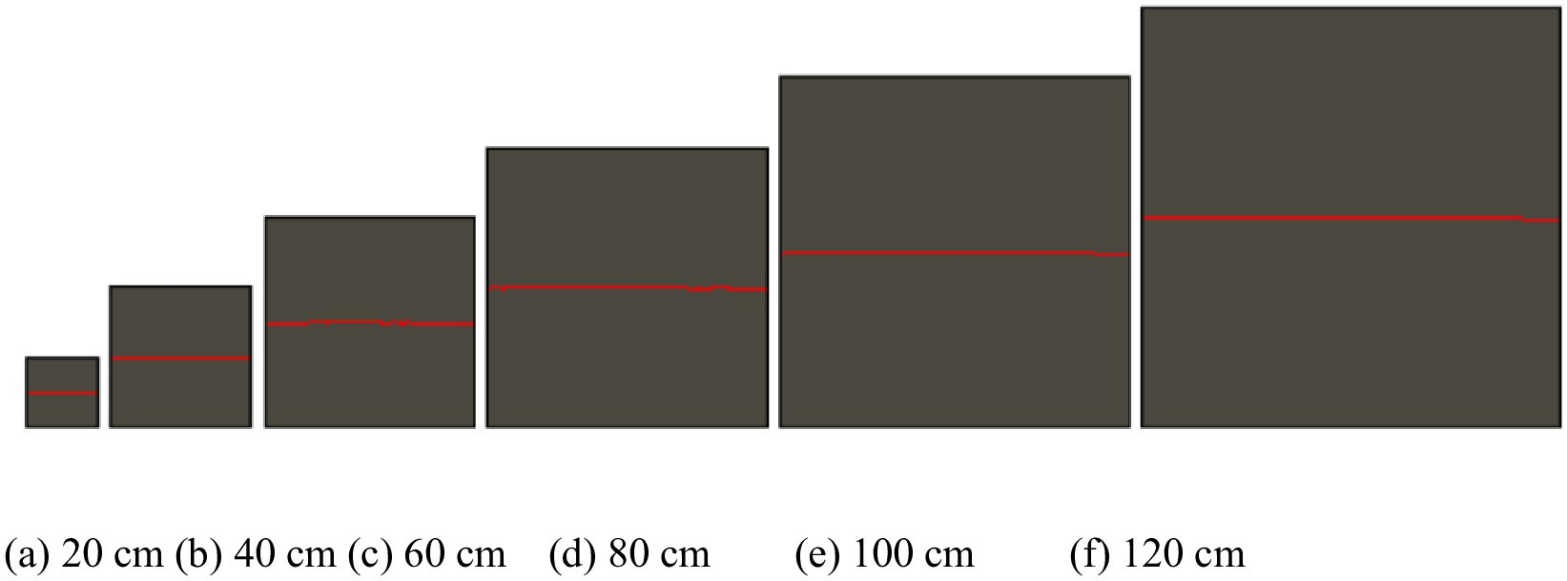
Rock models of different sizes when the JRC is 2.7.

Thirty numerical simulation models were formulated in this study. Only the numerical simulation model (200 mm in size (Fig 1)) and another model with a JRC of 2.7 (Fig 2) were presented as examples due to limited space. Through these examples, the modeling process in which the JRC and size change was introduced.

Similarly, although 30 working conditions were involved, the length of this article is limited by only a series of simulation models with a size of 200 × 200 mm^2^ (Fig 1) and with a JRC of 2.7 (Fig 2). Through these examples, the model formulation process follows when the JRC and size change are presented.

### Boundary conditions and rock structural plane parameters

#### Rock mechanical parameters and structural plane parameters

The main research object of this study was a rock with a continuous joint. The abovementioned samples of different sizes were input into the RFPA software. The deformation parameters of the rock and joints were as follows: the elastic moduli of the rock and structural plane were 8000 and 0.1 MPa, respectively. The Poisson’s ratio and internal friction angle (as a strength parameter) were 0.25 and 30°, respectively. The mechanical parameters of the rock and structural planes are summarized in Table 2.

**Table 2:** Rock mechanical parameters.

Material	Elastic Modulus (MPa)	Compressive strength (MPa)	Poisson’s ratio	Friction angle (°)
**Rock**	8000	60	0.25	30
Joint	1.1	1.5	0.30	30

#### Boundary conditions and loading methods

**Fig 3:**
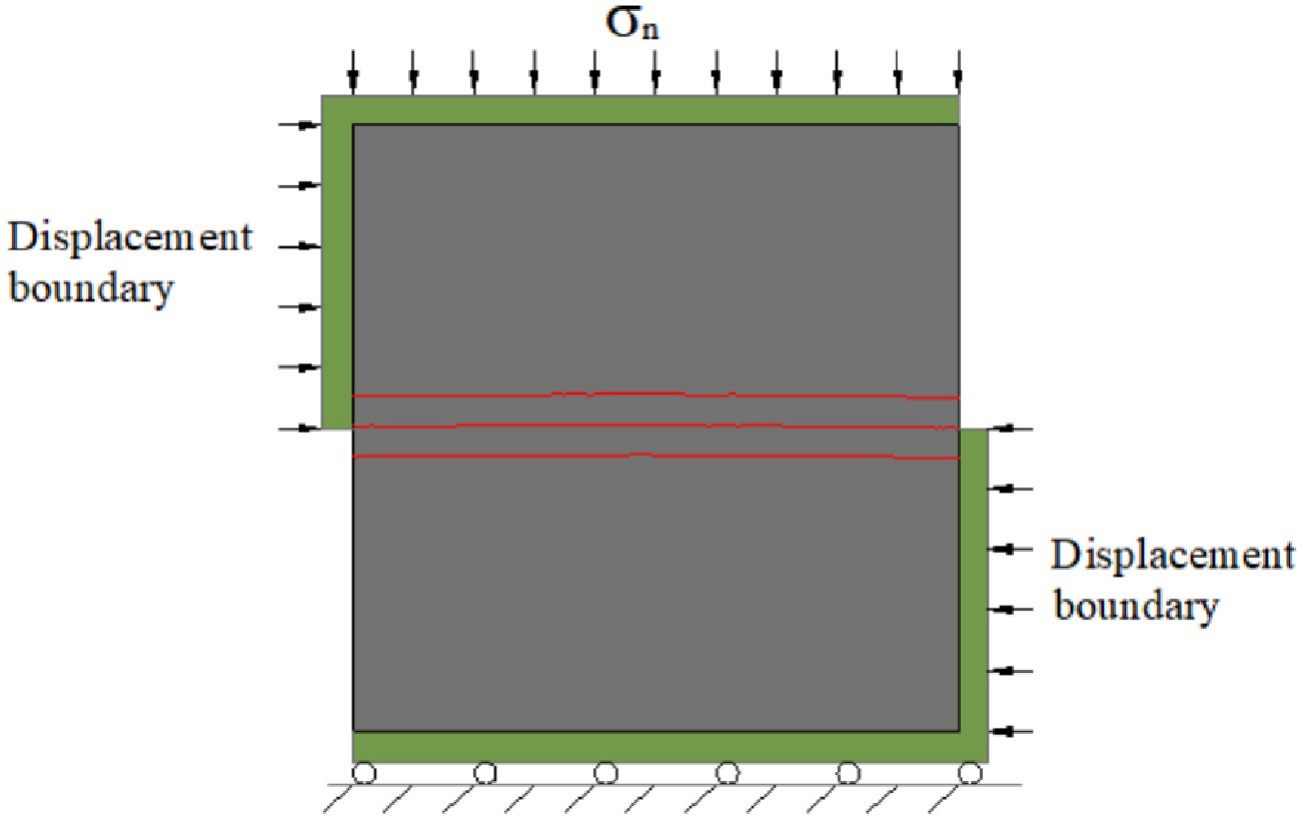
Load boundary of direct shear numerical model.

The theoretical basis of the simulation was the rock shear deformation theory. The mechanical model of the simulation test was a plane stress model, and the constraint condition was that the two sides of the model were subjected to horizontal shear, the upper side was subjected to load, and the lower side was restrained by the rolling support. The loading method adopted was displacement loading on both sides of the model. The initial loading of the model was zero, and the loading increment was 0.01 mm. During the simulation, the normal stress on the upper and lower planes of the model remained constant. The loading boundary of the numerical model is illustrated in Fig 3.

## Numerical simulation results and analysis

### Influence of JRC on shear strength of structural planes

The relationship between stress–strain and JRC was analyzed according to the simulation program and results of the first research. A method for fitting the relationship between the shear strength of structural planes and JRC was proposed. Moreover, a mathematical model is established for the two.

### Stress–strain curve analysis with different JRC coefficients

**Fig 4:**
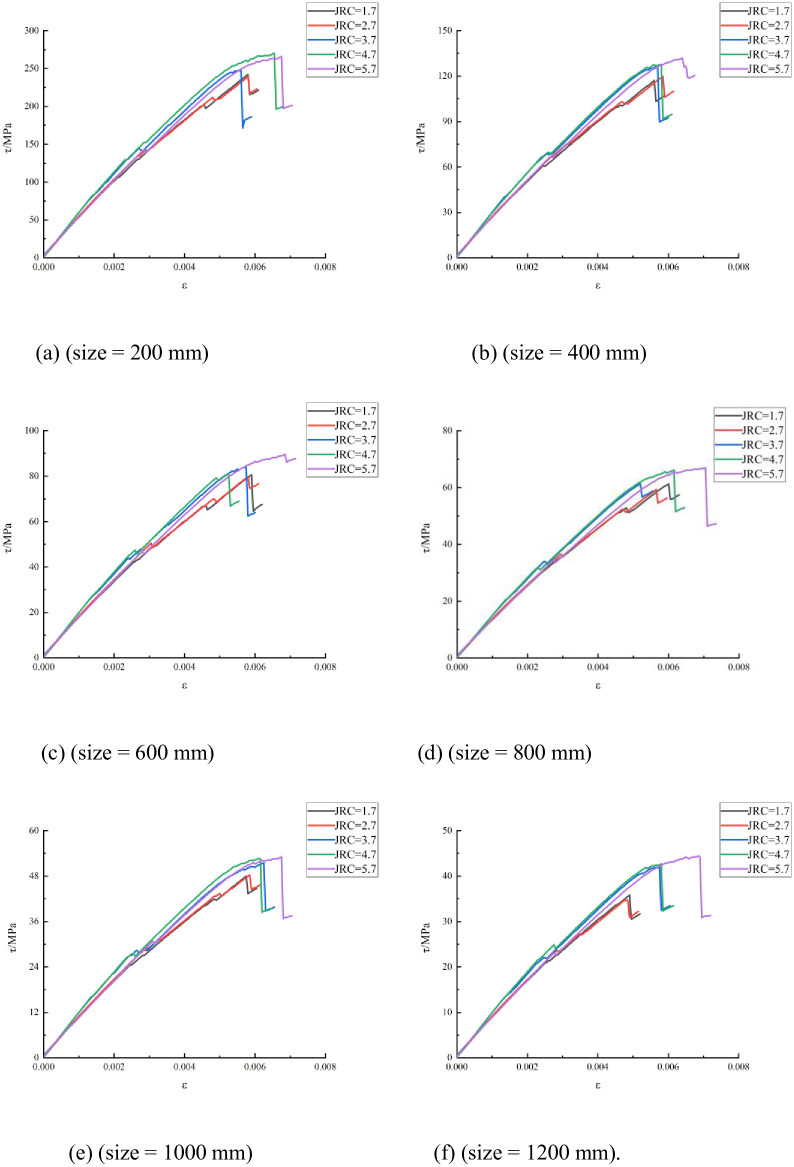
Stress–strain curves with different JRCs and sizes.

The numerical simulation results under each working condition were output for each simulation program, and the stress–strain curve under each working condition was plotted; the same coordinate system was used for all curves. Summary diagrams of the obtained stress–strain curves considering different JRCs for each simulation program are shown in Fig 4.

The shear strength of structural planes was explored based on the stress–strain curve under each working condition, as summarized in Table 3.

**Table 3:** Shear strength of structural plane with different JRCs.

Simulation Program	Size of structural plane (mm)	Shear strength of structural plane with different JRCs (MPa)				
		Program 7	Program 8	Program 9	Program 10	Program 11
		JRC = 1.7	JRC = 2.7	JRC = 3.7	JRC = 4.7	JRC = 5.7
**Program 1**	*l* = 200	242.13	239.01	247.79	269.79	265.98
**Program 2**	*l* = 400	117.05	119.89	126.15	127.8	131.95
**Program 3**	*l* = 600	80.58	79.67	83.82	81.1	89.25
**Program 4**	*l* = 800	61.29	59.31	61.34	66.24	66.95
**Program 5**	*l* = 1000	48	48.34	51.48	52.62	53.12
**Program 6**	*l* = 1200	35.81	34.7	41.99	42.7	44.24

Assuming that the curves follow the same law, only one of the graphs is selected as an example. The curve with a JRC of 5.7, shown in Fig 4(f), was selected to analyze the stress–strain trend. When the strain is in the range of 0–0.006, the shear stress increases linearly with the shear strain, and the deformation of the rock is in the elastic stage. When the strain is in the range of 0.006–0.0069, the shear stress changes from a linear increase to a nonlinear increase. As the strain increases, the maximum shear stress is 44 MPa, which then rapidly decreased from 44 to 31 MPa (i.e., a 31% decrease).

The analysis of the influence of JRC on the shear strength of structural planes is based on Fig 4(f). When the structural plane is 1200 mm in size, the strengths corresponding to the JRCs do not vary considerably in the elastic deformation stage. As the strain increases with the JRC, the shear strength of the structural plane also increases. The peak shear strength when the JRC is 5.7 exceeds that when it is 1.7. The main reason for this is that as the JRC increases, the degree of undulation increases. The stronger the cutting effect of shear, the greater is the elastic potential energy of the convex volume. When the protrusions on the plane of the crack are sheared, more energy is released, and the peak shear strength increases. In the nonlinear deformation stage, the shear stress change trend is affected by the JRC. The higher the JRC, the greater is the tendency of the shear stress to change.

Next, the influence of size on the shear strength of the structural plane is analyzed. The statistics summarized in Table 3 indicate that when the JRC varies from 1.7 to 5.7 and the rock size increases, the peak strength of the structural plane exhibits a downward trend. Moreover, when the rock size increases, the shear resistance and shear strength of the structural plane decreases. For example, as the size increases beyond 1200 mm, the shear strength of the structural plane diminishes, owing to the influence of the JRC.

Similarly, when the rock size is constant, but the JRC increases, the shear strength of the structural plane increases. When the JRC is constant, but the rock size increases, the shear strength of the structural plane shows a decreasing trend.

### Fitting method of the relationship between shear strength and JRC

**Fig 5:**
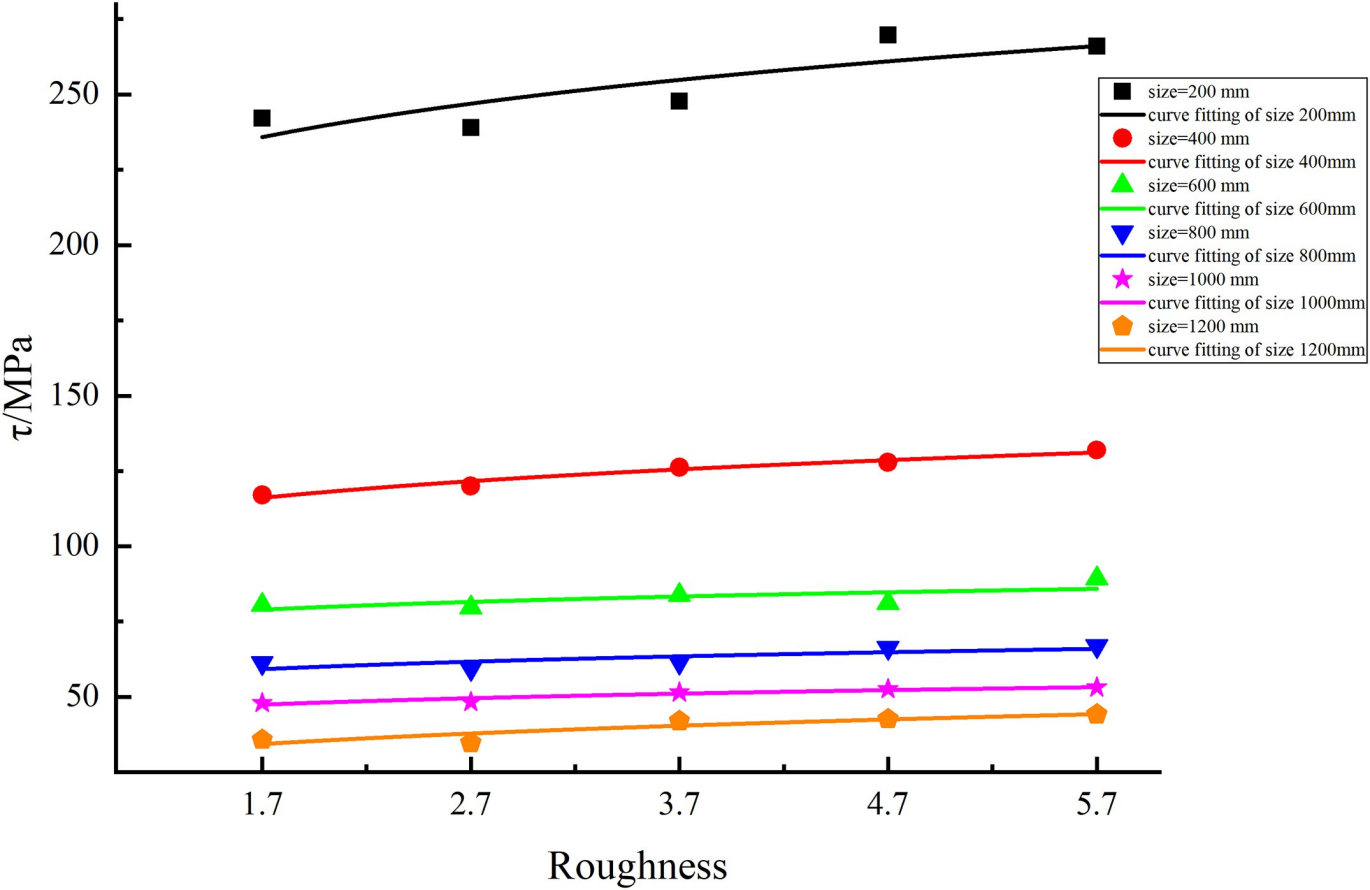
Fitting curves of shear strength with different JRCs.

The statistics listed in Table 3 show that as the JRC increases, the shear strength of the structural planes gradually increases. The data were imported into Origin software to draw a scatter plot of the shear strength and JRC of each numerical simulation program. The fitting curve of the JRC and shear strength was drawn based on the scatter diagram, as shown in Fig 5.

The fitting curves in Fig 5 indicate that the shear strength of structural planes gradually increases when the JRC increases; however, the size remains the same. Moreover, despite the different sizes of structural planes, the law of change is similar. The fitting curve in Fig 5 shows that the relationship between the JRC and shear strength at different sizes can be derived, as listed in Table 4.

**Table 4:** Fitting relationship between shear strength and JRCs.

Size of structural plane (mm)	Fitting formula	R^2^
**200**	*τ*(*r*) = 223.66*r*^0.078^	0.901
**400**	*τ*(*r*) = 110.02*r*^0.087^	0.963
**600**	*τ*(*r*) = 76.1*r*^0.094^	0.891
**800**	*τ*(*r*) = 56.45*r*^0.105^	0.910
**1000**	*τ*(*r*) = 45.08*r*^0.113^	0.911
**1200**	*τ*(*r*) = 30.74*r*^0.14^	0.913

In the list, when the JRC is *r*, *τ*(*r*) is the shear strength of the structural planes (unit: MPa).

The fitting relationship indicates that the shear strength and JRC have a satisfactory fit, providing a means for quantitatively analyzing them for engineering practice.

### Formulation of mathematical model of shear strength and JRC

**Fig 6:**
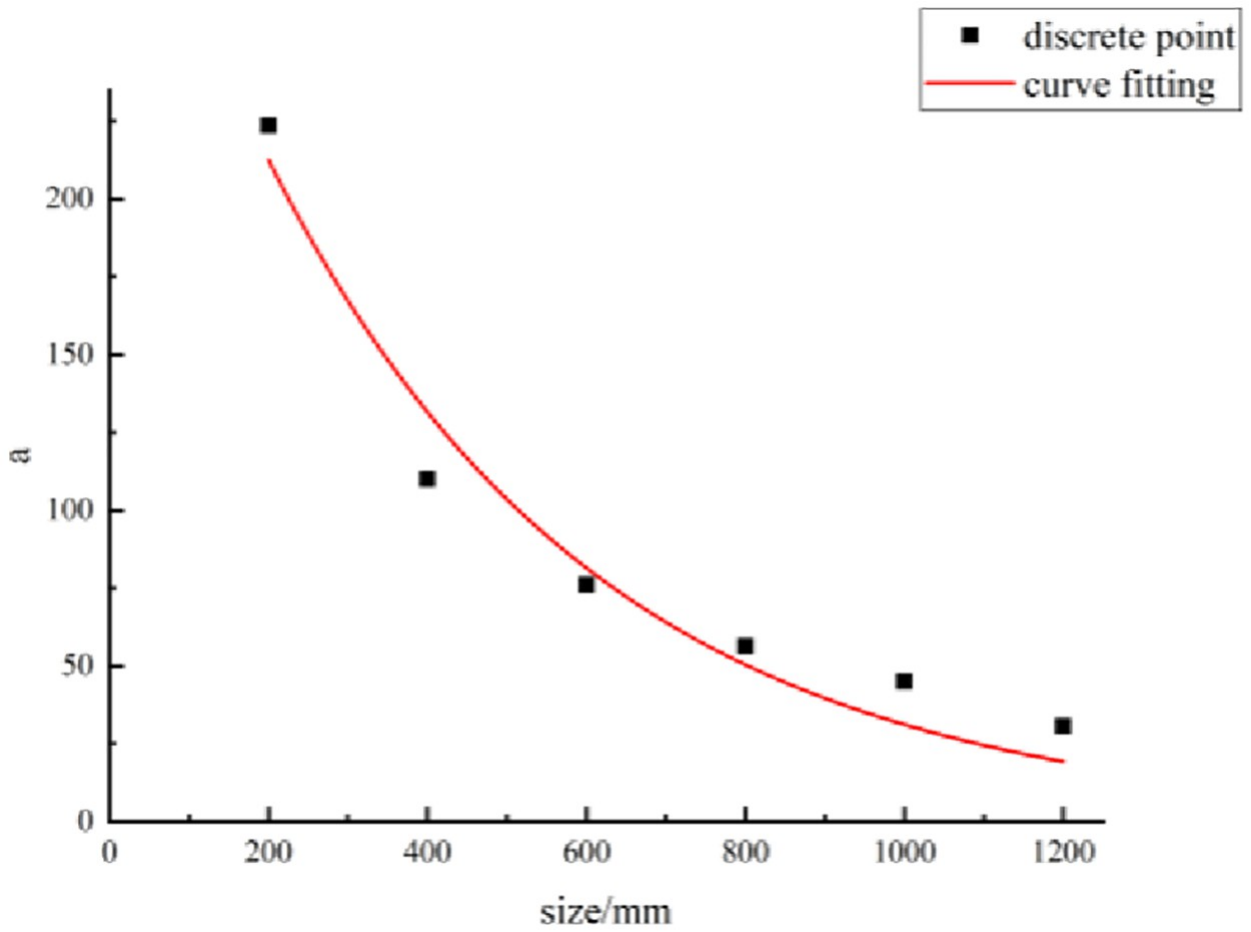
Fitting curve of parameter a.

**Fig 7:**
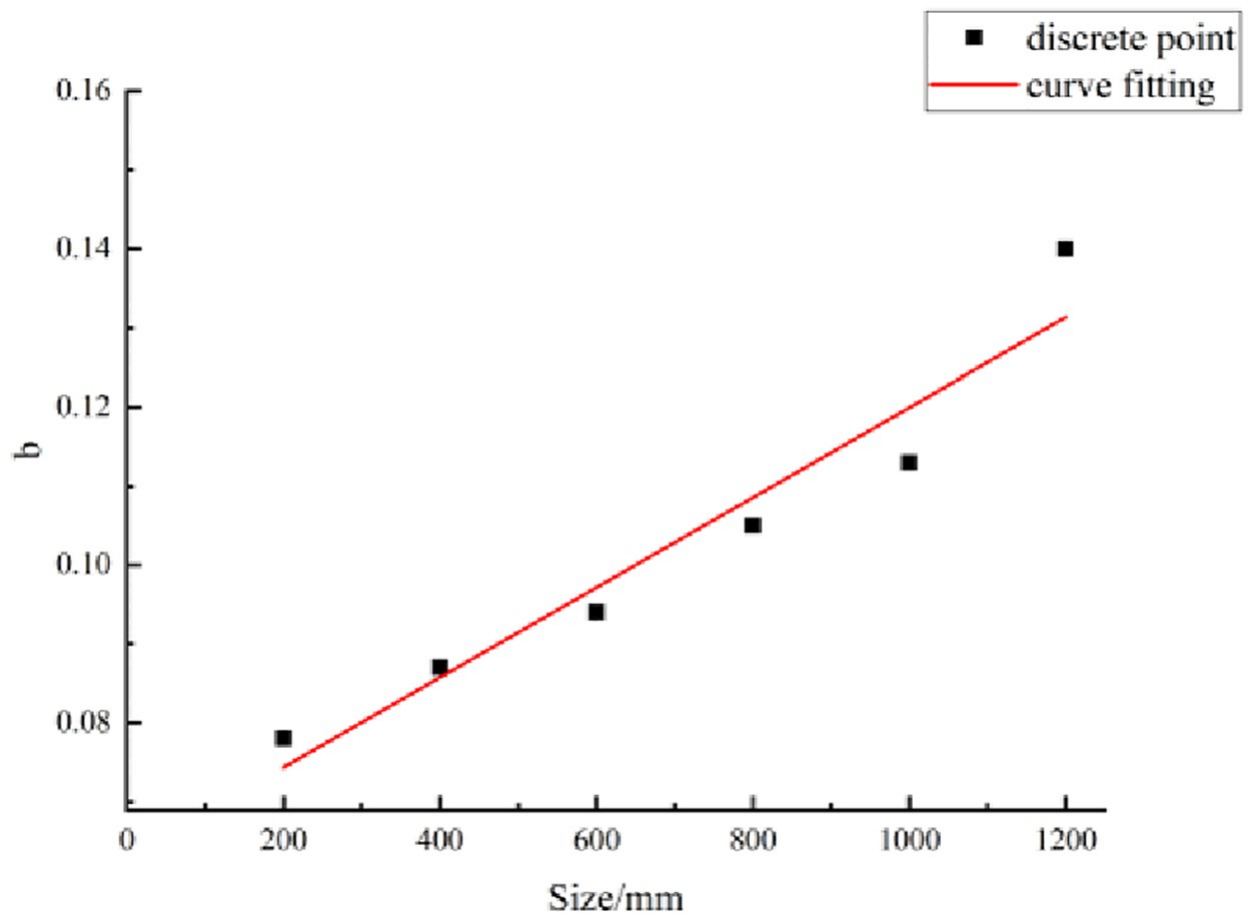
Fitting curve of parameter b.

(a) Mathematical model (1)
  The function types of the formulas were analyzed according to the relationship between the shear strength of the structural planes and JRC at different sizes. A mathematical model for the shear strength versus JRC is proposed as follows:
$$\tau \left(r\right)=ar^{b},$$ (2)
  where *τ(r)* is the shear strength of the structural plane when the JRC is *r* (unit: MPa), and *a* and *b* are parameters.
  The relationship between the shear strength and JRC is given by Eq (2), which contains parameters *a* and *b*. If *a* and *b* have been determined, the shear strength corresponding to any JRC can be obtained.
(b) Method for parameter evaluation
  The statistics listed in Table 4 show that the parameter values are related to the size of the structural planes. The size of the structural plane and parameter values were considered as the abscissa and ordinate, respectively, to plot their scatter diagram. Then, based on this scatter diagram, parameters *a* and *b* and the size of the structural planes are fitted, as shown in Figs 6 and 7.
  According to the fitting curves in these figures, the relationships between parameters *a* and *b* and the size of the structural planes are as follows:
$$a=343.47e^{-0.0024l},$$ (3)
$$b=0.06293+5.7\times 10^{-5}l.$$ (4)
(c) Mathematical model (2)
  The mathematical model of the parameters and the size of the structural planes were substituted into the mathematical model of the shear strength of the structural planes and JRC. Accordingly, the mathematical model of the shear strength and JRC is derived as follows:
$$\tau \left(r\right)=343.47e^{-0.0024l}r^{\left(0.06293+5.7\times 10^{-5}l\right)},$$ (5)
  where *τ*(*r*) is the shear strength of the structural plane when the JRC is *r* (unit: MPa), and *l* is the size of the structural plane (unit: mm).

The derived mathematical model of the shear strength of structural planes and the JRC can be applied to a field rock with structural planes. It provides guidance and a method for the quantitative analysis of JRCs and shear strengths of structural planes for engineering practice.

### Influence of size on shear strength of structural planes

#### Stress–strain curve analysis considering different sizes

**Fig 8:**
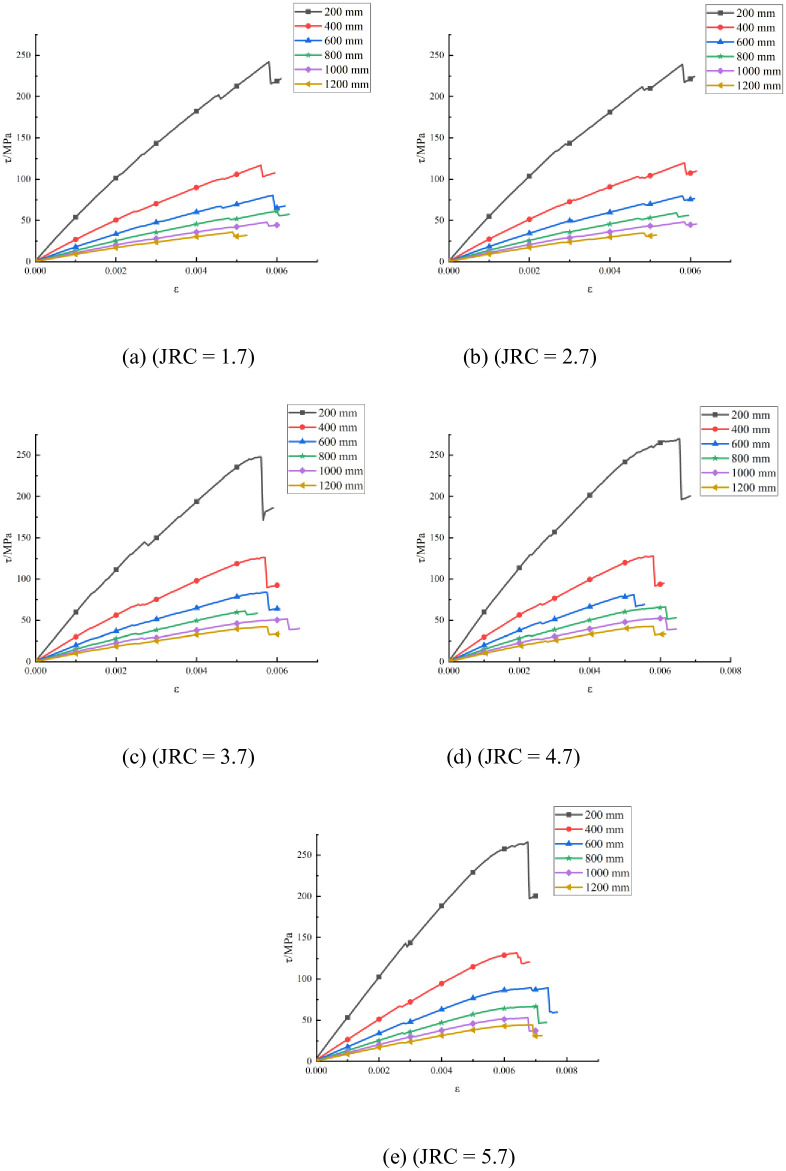
Stress–strain curves with different structural plane sizes and JRCs.

The numerical simulation results under each working condition were output for each simulation program, and a stress–strain curve was obtained. The stress–strain curves in each simulation program under any working conditions were drawn using the same coordinate system. A summary of the stress-strain curves corresponding to different structural plane sizes for each simulation program was derived, as shown in Fig 8.

The shear strength of the structural planes was explored according to the stress–strain curves under each working condition in the numerical simulation program. The shear strengths under all the working conditions are summarized in Table 5.

**Table 5:** Shear strength at different sizes of structural plane.

SimulationProgram	Joint Roughness coefficient	Shear strength at different sizes of structural plane (MPa)					
		Program 1	Program 2	Program 3	Program 4	Program 5	Program 6
		200 mm	400 mm	600 mm	800 mm	1000 mm	1200 mm
**Program 7**	1.7	242.13	117.05	80.58	61.29	48	35.81
**Program 8**	2.7	239.01	119.89	79.67	59.31	48.34	34.7
**Program 9**	3.7	247.79	126.15	83.82	61.34	51.48	41.99
**Program 10**	4.7	269.79	127.8	81.1	66.24	52.62	42.7
**Program 11**	5.7	265.98	131.95	89.25	66.95	53.12	44.24

Assuming that the curves follow the same law, only one of the graphs is selected as an example. Consider the structural plane size of 200 mm, shown in Fig 8(e) as an example to analyze the stress–strain trend. When the strain is small, the stress–strain curve is approximately a straight line, and the model is in the elastic deformation stage. After the stress reaches the peak strength of 265.98 MPa, the curve rapidly drops to 197.23 MPa; the stress fluctuates near this value as the strain increases.

The analysis of the influence of size on the shear strength of structural planes is also based on Fig 8(e) as an example. With increasing strain, when the JRC is 5.7, the larger the structural plane size, the lower the shear strength. The peak strength of the structural plane whose size is 200 mm is 265.98 MPa, whereas that whose size is 400 mm is 131.95 MPa; the peak strengths differ by 134.03 MPa. The peak strength of the structural plane whose size is 1000 mm is 53.12 MPa, whereas that whose size is 1200 mm is 44.24 MPa; the peak strength difference is only 8.88 MPa. This shows that as the size increases, the shear strength gradually decreases, indicating that the size affects the shear strength of a structure plane.

The influence of JRC on the shear strength of structural planes is analyzed next. The statistics summarized in Table 5 show that when the size is 200 mm and the JRC increases from 1.7 to 5.7, the shear strength increases from 242.13 to 265.98 MPa. When the model sizes were 400, 600, 800, 1000, and 1200 mm, the shear strength of the structural surfaces showed an increasing trend as the JRC increased because the greater the JRC, the higher the undulation. The upper and lower interfaces of the model are staggered, and more protruding bodies are damaged by shearing.

The above analysis shows that when the JRC is constant, the shear strength of the structural surfaces decreases as the size increases. When the size is constant, the shear strength of the structural planes increases as the JRC increases.

#### Fitting method of the relationship between the shear strength and size of structural planes

**Fig 9:**
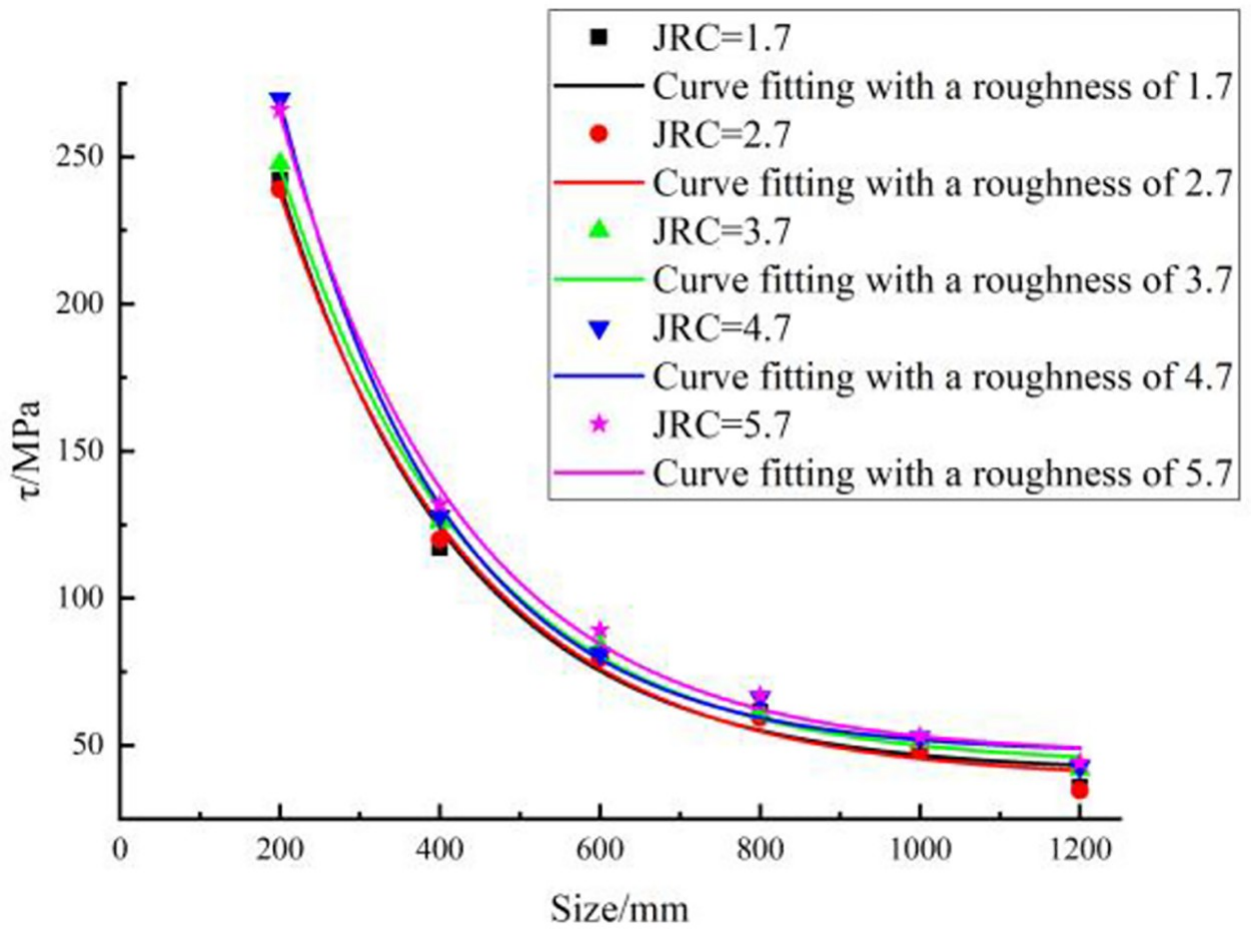
Fitting curves of shear strength with different sizes of a structural plane.

The statistics summarized in Table 5 indicate that as the size increases, the shear strength of the structural planes gradually decreases. The data were imported into Origin software to draw a scatter plot of the shear strength and size in each numerical simulation program. The fitting curve of the shear strength and size was drawn based on a scatter diagram, as shown in Fig 9.

The fitting curves in Fig 9 indicate that the shear strength of the structural planes gradually decreases as the size increases under the same JRC. The law of change for different JRCs was similar. The fitting relationship between the shear strength and size at different JRCs listed in Table 6 can be obtained based on the fitting curve in Fig 9.

**Table 6:** Fitting relationship between shear strength and size of structural plane.

JRC	Fitting formula	R^2^
**1.7**	$\tau \left(l\right)=40.73+480.36e^{\left(\frac{-l}{248.07}\right)}$	0.994
**2.7**	$\tau \left(l\right)=41.94+493.7e^{\left(\frac{-l}{240.68}\right)}$	0.996
**3.7**	$\tau \left(l\right)=43.26+503.26e^{\left(\frac{-l}{234.81}\right)}$	0.998
**4.7**	$\tau \left(l\right)=45.02+534.36e^{\left(\frac{-l}{217.9}\right)}$	0.997
**5.7**	$\tau \left(l\right)=46.48+549.94e^{\left(\frac{-l}{210.89}\right)}$	0.997

In the table, *τ*(*l*) is the shear strength of the structural plane when the size is *l* (unit: MPa), and *l* is the size of the structural plane (unit: mm).

The fitting relationship summarized in Table 6 shows that the shear strength and size have an acceptable fit, providing a means for quantitatively analyzing the size and shear strength of structural planes in engineering practice.

### Formulation of a mathematical model of shear strength and size of structural planes

**Fig 10:**
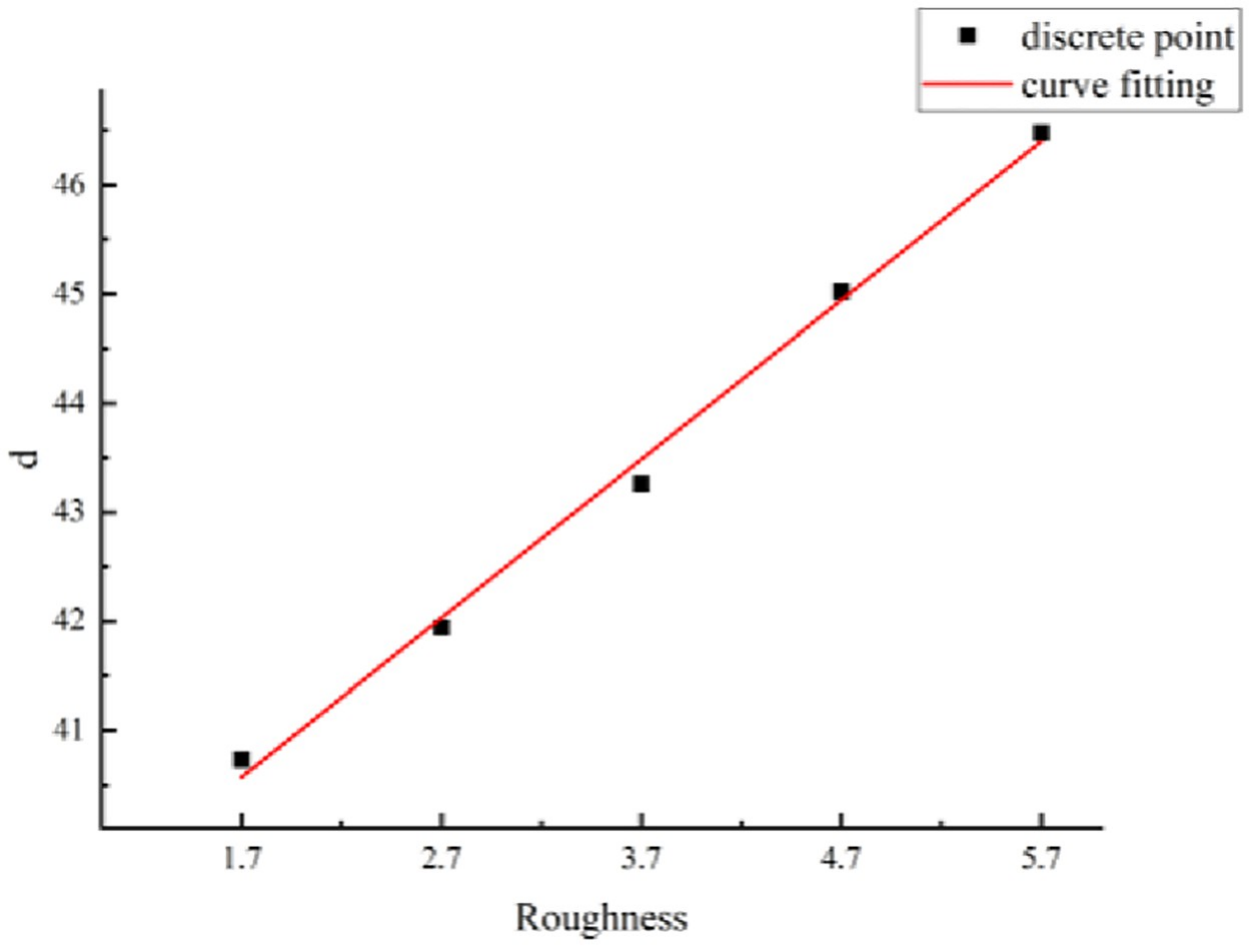
Fitting curve of parameter d.

**Fig 11:**
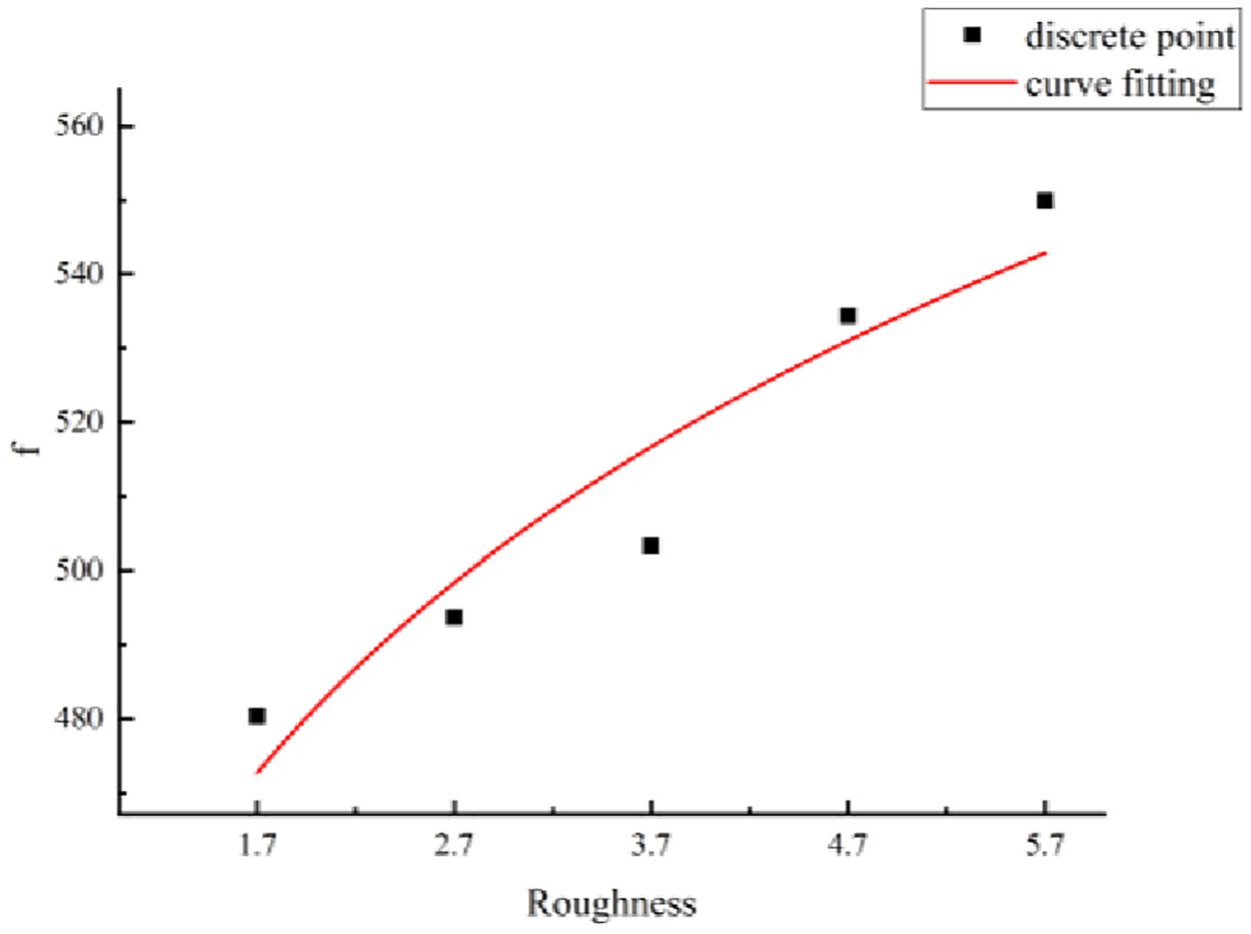
Fitting curve of parameter f.

**Fig 12:**
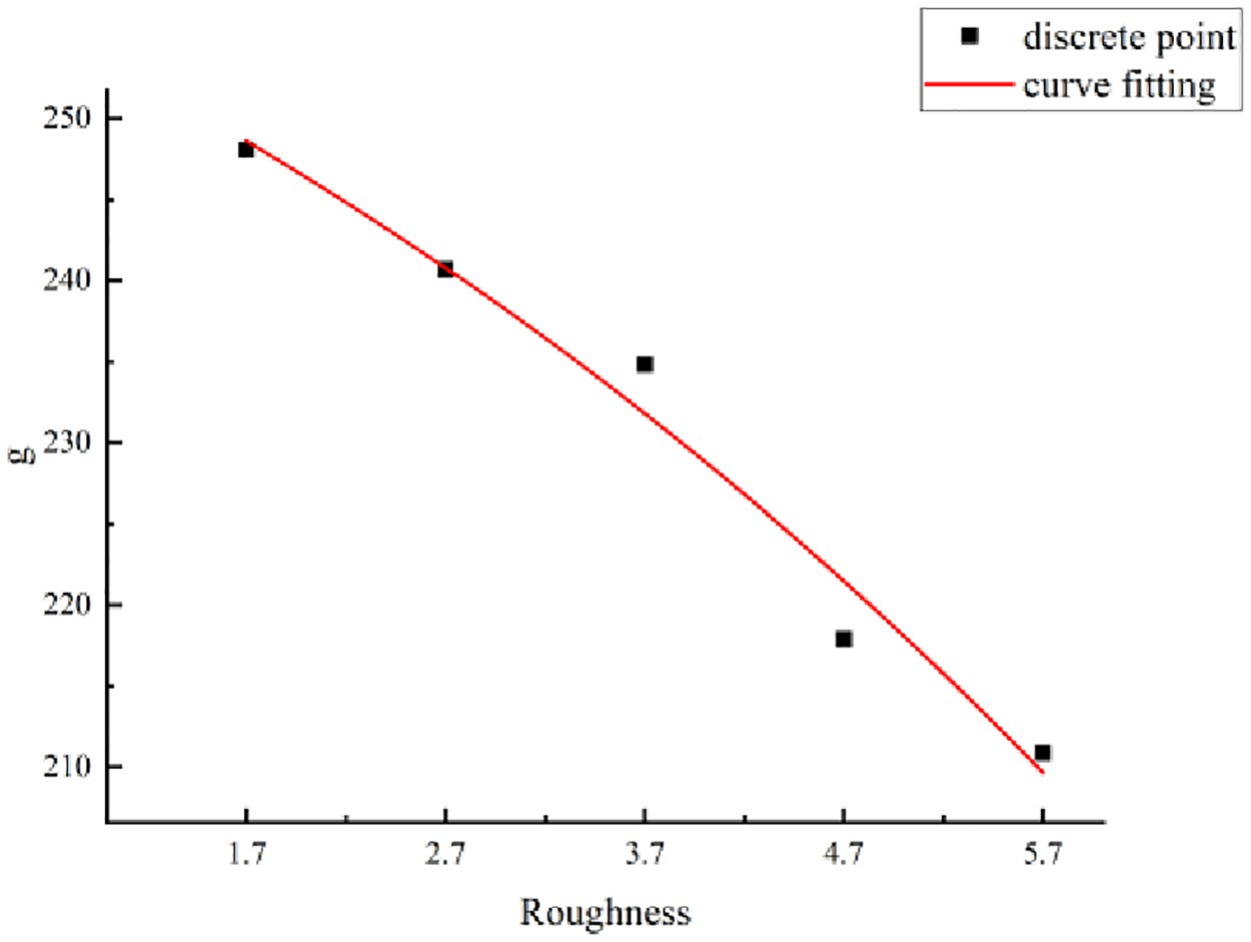
Fitting curve of parameter g.

(a) Mathematical model (3)
  The function types of the formulas were analyzed based on the relationship between the shear strength of structural planes and the size considering different JRCs. A mathematical model for the shear strength of the structural planes and size is proposed as follows:
$$\tau \left(l\right)=d+fe^{\left(\frac{-l}{g}\right)},$$ (6)
  where *τ*(*l*) is the shear strength of the structural plane of size *l* (unit: MPa), *l* is the size of the structural plane (unit: mm), and *d*, *f*, and *g* are parameters.
  The relationship between the shear strength of the structural planes and the size is given by Eq (6), which includes the parameters *d*, *f*, and *g*. When *d*, *f*, and *g* are determined, the shear strength of the structural planes considering any size can be derived.
(b) Method for parameter evaluation.
  The statistics summarized in Table 6 indicate that the parameter values are related to the JRC. Based on the data listed in this table, the JRC and parameters were considered as the abscissa and ordinate, respectively, to draw a scatter diagram. Based on this diagram, the relationship between the parameters (i.e., *d*, *f*, and *g*) and JRC are fitted, as shown in Figs 10–12.
  According to the fitting curves shown in Figs 10–12, the relationships between parameters *d*, *f*, and *g* and the size of the structural planes are as follows:
d=38.09+1.458r, (7)
$$f=444.84r^{0.114},$$ (8)
$$g=302.08-42.37e^{\frac{r}{7.31}}.$$ (9)
(c) Mathematical model (4)
  The mathematical model of the JRC and parameters was substituted into the mathematical model of the shear strength of the structural planes and size. Accordingly, a mathematical model of the shear strength of the structural planes and size is derived as follows:
$$\tau \left(l\right)=\left(38.0914+1.458\text{r}\right)+\left(444.84r^{0.114}\right)e^{\left(\frac{-l}{302.08-42.37e^{\frac{r}{7.31}}}\right)},$$ (10)
  where *τ(l)* is the shear strength of the structural plane of size *l* (unit: MPa), *r* is the JRC, and *l* is the size of the structural plane (unit: mm).

The derived mathematical model of the shear strength of structural planes and size can be extended to field rocks containing structural planes. It provides guidance and a method for quantitatively analyzing the size and shear strength of structural planes for engineering practice.

### Formulation of the mathematical model of the JRC and characteristic size and strength of structural planes

As the sizes of the structural planes and the number of basic units increase, the shear strength value of structural planes tends to stabilize, which is the characteristic strength value of structural planes. At this strength, the related size is the characteristic size of the structural planes.

The exact characteristic size is difficult to assess quantitatively. The absolute value of the slope of the curve was derived using the two sides of Eq (5), using the following equation:

$$\tau^{\prime }\left(l\right)=-\frac{f}{g}e^{\left(\frac{-l}{g}\right)}.$$ (11)

Formulate

$$\left|\tau^{\prime }\left(l\right)\right|\leq \alpha .$$ (12)

to obtain

$$l\geq g\;\text{ln}\;\frac{f}{g}-g\;\text{ln}\;\alpha ,$$ (13)

where α is the acceptable absolute value of the slope, which can be an extremely small value.

Eq (13) may be regarded as yielding the characteristic size by substituting the fitting parameters into it. Assuming that *α* = 0.05, the characteristic size of each structural plane can be obtained. The relationship between the characteristic size and JRC can be obtained by calculation, as summarized in Table 7.

**Table 7:** Relationship between characteristic size and JRC.

JRC	1.7	2.7	3.7	4.7	5.7
**Shear characteristic size (mm)**	904.48	896.37	880.05	857.05	827.43

The statistics listed in Table 7 show that as the JRC gradually increases, the characteristic size of the structural planes gradually decreases. The relationship between the characteristic size and JRC can be analyzed qualitatively.

**Fig 13:**
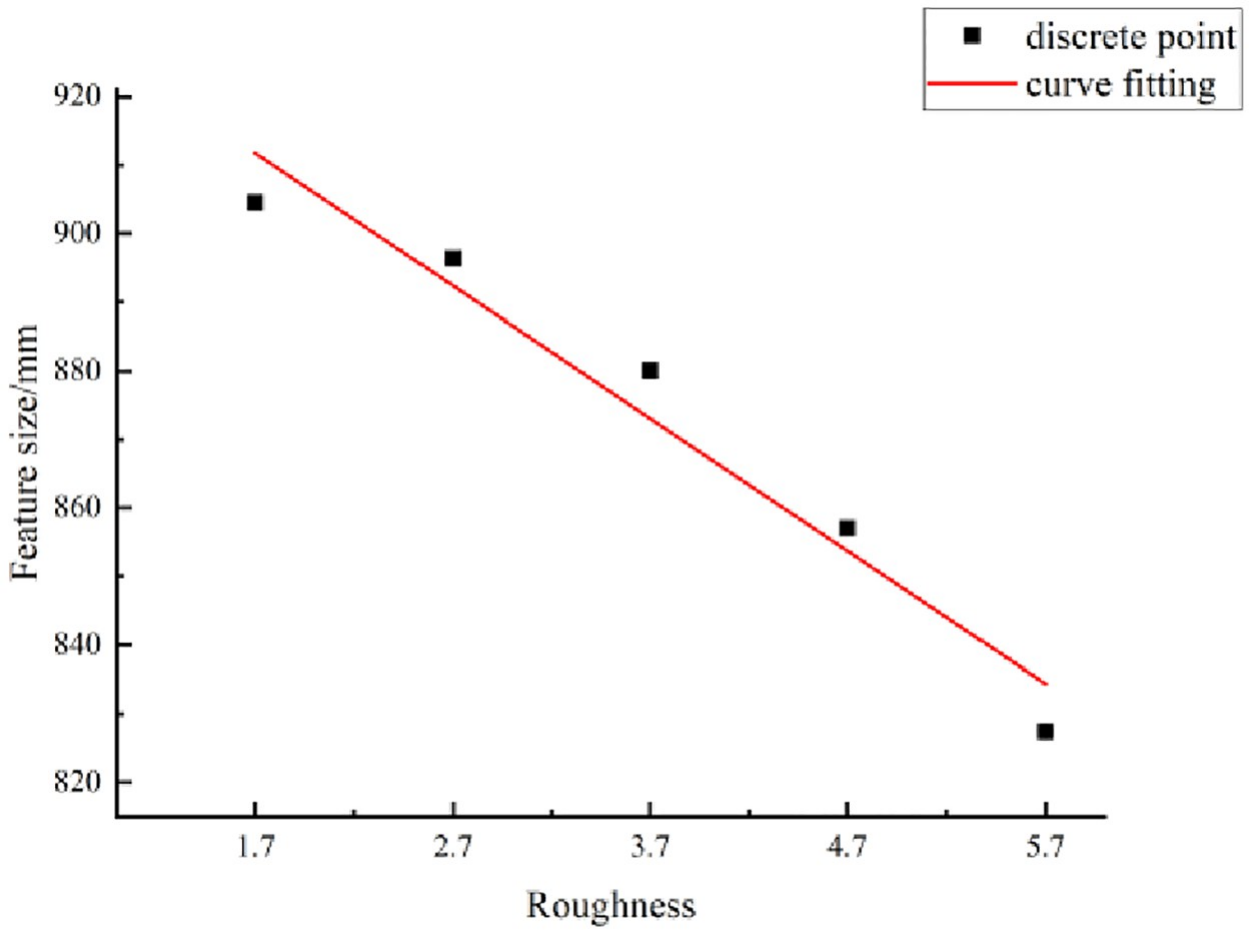
Characteristic sizes of structural plane with different JRCs.

The JRC and characteristic size were considered as the abscissa and ordinate, respectively, to draw a scatter plot. The fitting curve was drawn based on a scatter plot, as shown in Fig 13.

A quantitative description of the size effect is depicted in terms of the characteristic size. The fitting curve in Fig 13 shows that the characteristic size of the structural planes exhibits a decreasing trend when the JRC increases.

This curve conforms to a linear relationship. A mathematical model for the characteristic size of the structural planes and the JRC is as follows:

D=944.64−19.34r, (14)

where *D* is the characteristic size (mm) and *r* is the JRC.

Eq (14) provides a quantitative description of the relationship between the JRC and the characteristic size of the structural planes. If the JRC can be measured, then the characteristic size, essential for engineering applications, can be obtained accurately using Eq (14). Considering the influence of the size effect, when the shear strength of the structural surface needs to be calculated, only the rocks larger than the characteristic size need to be selected for testing.

### Formulation of the mathematical model of the JRC and shear characteristic strength of structural planes

According to Eq (10), the characteristic strength of the structural planes can be obtained based on the characteristic size. The calculated relationship between the characteristic shear strength and JRC is summarized in Table 8.

**Table 8:** Relationship between characteristic shear strength and JRC.

JRC	1.7	2.7	3.7	4.7	5.7
**Characteristic shear strength (MPa)**	53.00	54.07	55.07	56.01	56.88

The statistics listed in Table 8 show that as the JRC increases, the characteristic shear strength of the structural planes gradually increases. The relationship between the characteristic strength and JRC can be analyzed qualitatively.

**Fig 14:**
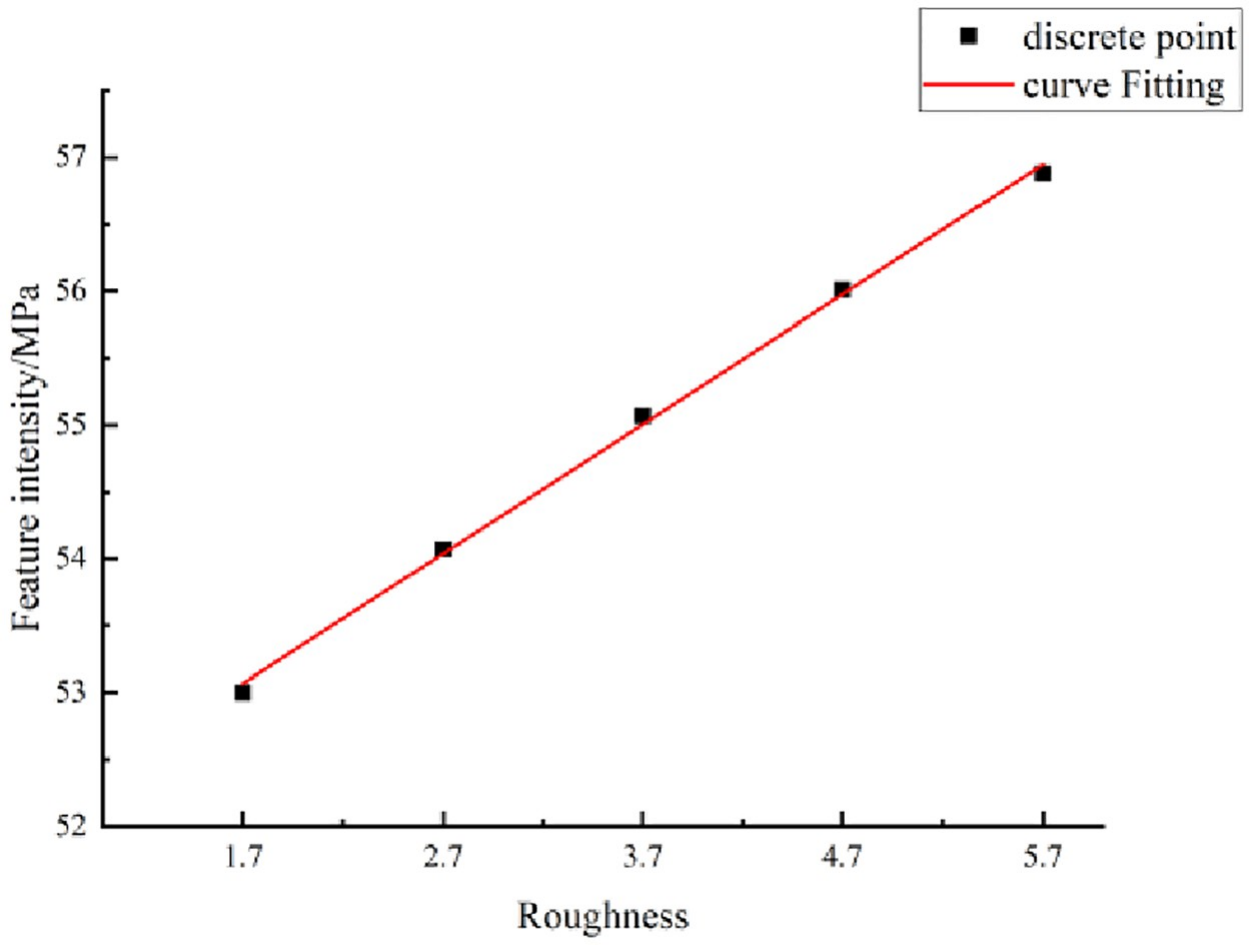
Characteristic shear strength of structural plane with different JRCs.

The JRC and characteristic strength were considered as the abscissa and ordinate, respectively, for drawing a scatter diagram. The fitting curve was drawn based on a scatter plot, as shown in Fig 14.

Fig 14 shows that the characteristic shear strength of the structural planes tends to increase with JRC.

This curve conforms to a linear relationship. A mathematical model of the characteristic shear strength of the structural planes and the JRC is

$$\tau_{w}=51.417+0.97r,$$ (15)

where *τ*_*w*_ is the characteristic shear strength (MPa) and *r* is the JRC.

Eq (15) provides a quantitative description of the relationship between the characteristic shear strength of the structural planes and the JRC. If the JRC can be measured, the characteristic shear strength, essential for engineering applications, can be obtained accurately using Eq (15).

## Discussion

The JRC and size affect the shear strength, characteristic size, and shear strength of structural planes; however, this relationship is yet to be obtained. This study establishes four mathematical relationships: (i) shear strength of structural planes and JRC, (ii) shear strength of structural planes and size, (iii) characteristic size of structural planes and JRC, (iv) characteristic shear strength of structural planes and JRC. Their innovations are described below. By establishing four the mathematical models, the relationships between the shear strength, the characteristic size of the structural plane, the characteristic shear strength of the structural plane, and JRC were quantified. Furthermore, the relationship between shear strength and size was quantified; this relationship has important scientific significance. Once the JRC and size of the structural plane are obtained, the shear strength, characteristic size, and characteristic shear strength can be easily obtained. They provide considerable engineering guidance. Below, we list the differences between them and consider existing research from the perspective of mathematical relationships.

(i) Relationship between the shear strength of structural planes and JRC
  First, the general form of the mathematical relationship between the shear strength of structural planes and the JRC is given by considering different rock sizes and JRC. Then, combined with the change in rock size, the specific form of the mathematical relationship is given. In the existing research, few scholars have considered the role of size in discussing the influence of JRC on the shear strength of structural planes. The relationship between the shear strength of structural planes, JRC, and rock size has not yet been established.
(ii) Relationship between the shear strength of structural planes and size
  First, the general mathematical relationship between the shear strength of structural planes and the size is given by considering the JRC and rock sizes. The specific form of the mathematical relationship is then combined with the change in the JRC. In the existing research on the size effect of the shear strength of structural planes, few scholars have considered the influence of JRC on it. The relationship between the shear strength of structural planes, rock size, and JRC has not yet been established.
(iii) Relationship between the characteristic size of structural planes and JRC
  The relationship between the characteristic size of structural planes and JRC is based on relationship between the shear strength of structural planes and JRC. Presently, no scholars have explored their relationship.
(iv) Relationship between the characteristic shear strength of structural planes and JRC
  The relationship between the characteristic shear strength of structural planes and JRC is based on relationships (i) and (iii). In existing research, no scholars have established the relationship yet.

Many factors affect the shear strength of structural planes, but few studies have combined roughness and rock size to study the shear strength. Relational expressions (i) and (ii) consider the changes in roughness and rock size simultaneously, have certain innovation and engineering guiding values.

The relational expressions (iii) and (iv) all consider the influence of roughness on them and establish the relationship between roughness and them. Thus, when the roughness is known, the corresponding characteristic size and characteristic strength can be obtained through these relational formulas. At the engineering site, it has good guiding significance.

However, this study had some limitations. Because this research was performed using numerical simulations, the simulation conditions were simplified. When the numerical models were formulated, the model only had one structural plane, and the effects of small cracks and water content, that may be present in the rock mass, are neglected. The factors considered were incomplete, and the study could be improved by including more factors. Moreover, when the research is employed at an engineering site, it can only be applied to specific rock masses with structural planes.

## Conclusion

This study investigated the influence of roughness and size on the shear strength of a structural plane. The stress–strain curves of the shear strengths of different roughness and sizes were investigated numerically. From these simulations, the following conclusions can be drawn:

1. The shear strength of the structural plane is related to the roughness and obeys the power function distribution, which conforms to the following relationship:
$$\tau \left(r\right)=ar^{b},$$
  and our simulations obtained
$$\tau \left(r\right)=343.47e^{-0.0024l}r^{\left(0.06293+5.7\times 10^{-5}l\right)}.$$
2. The shear strength of the structural plane was found to be related to the size and obeys the exponential function distribution, which conformed to the following relationship:
$$\tau \left(l\right)=\text{d}+\text{f}e^{\left(\frac{-l}{g}\right)},$$
  and our simulations obtained
$$\tau \left(l\right)=\left(38.0914+1.458\text{r}\right)+\left(444.84r^{0.114}\right)e^{\left(\frac{-l}{302.08-42.37e^{\frac{r}{7.31}}}\right)}.$$
3. The characteristic size of the shear strength of the structural plane was found to be related to the roughness, which varied linearly with the JRC. Our simulation provided the following form:
D=944.64−19.34r.
4. The characteristic shear strength of the structural plane was found to be related to the roughness, which varied linearly with the JRC. Our simulation gives the particular form:
$$\tau_{w}=51.417+0.97r.$$
